# Omicron subvariant BA.5 efficiently infects lung cells

**DOI:** 10.1038/s41467-023-39147-4

**Published:** 2023-06-13

**Authors:** Markus Hoffmann, Lok-Yin Roy Wong, Prerna Arora, Lu Zhang, Cheila Rocha, Abby Odle, Inga Nehlmeier, Amy Kempf, Anja Richter, Nico Joel Halwe, Jacob Schön, Lorenz Ulrich, Donata Hoffmann, Martin Beer, Christian Drosten, Stanley Perlman, Stefan Pöhlmann

**Affiliations:** 1 Infection Biology Unit, German Primate Center – Leibniz Institute for Primate Research, Göttingen, Germany; 2 Faculty of Biology and Psychology, Georg-August-University Göttingen, Göttingen, Germany; 3 Departments of Microbiology and Immunology, BSB 3-712, University of Iowa, Iowa City, IA USA; 4 Institute of Virology, Charité - Universitätsmedizin Berlin, Campus Charité Mitte, Berlin, Germany; 5 Institut für Virusdiagnostik (IVD), Friedrich-Loeffler-Institut, Greifswald - Insel Riems, Germany

**Keywords:** SARS-CoV-2, Virus-host interactions

## Abstract

The SARS-CoV-2 Omicron subvariants BA.1 and BA.2 exhibit reduced lung cell infection relative to previously circulating SARS-CoV-2 variants, which may account for their reduced pathogenicity. However, it is unclear whether lung cell infection by BA.5, which displaced these variants, remains attenuated. Here, we show that the spike (S) protein of BA.5 exhibits increased cleavage at the S1/S2 site and drives cell-cell fusion and lung cell entry with higher efficiency than its counterparts from BA.1 and BA.2. Increased lung cell entry depends on mutation H69Δ/V70Δ and is associated with efficient replication of BA.5 in cultured lung cells. Further, BA.5 replicates in the lungs of female Balb/c mice and the nasal cavity of female ferrets with much higher efficiency than BA.1. These results suggest that BA.5 has acquired the ability to efficiently infect lung cells, a prerequisite for causing severe disease, suggesting that evolution of Omicron subvariants can result in partial loss of attenuation.

## Abstract (web summary)

The Omicron variant is partially attenuated, likely because it fails to efficiently infect lung cells. Here, Hoffmann et. al. show that this defect can be lost during Omicron evolution as demonstrated for the subvariant BA.5 that robustly infects lung cells in vitro and in vivo.

## Introduction

The SARS-CoV-2 Omicron variant (PANGO lineage B.1.1.529 and its sublineages) emerged in winter 2021 from a so far unknown reservoir, potentially immunocompromised patients, and became rapidly globally dominant. The coronavirus spike (S) protein mediates viral entry into host cells and is the major target of neutralizing antibodies. The Omicron variant harbors more than 30 mutations in the viral S protein that are associated with an unprecedented level of evasion of neutralizing antibodies induced upon infection and vaccination^1–5^. As a consequence, the Omicron variant can spread efficiently in populations with a certain level of preexisting immunity. However, its capacity to cause disease is reduced as compared to previously circulating variants^6,7^.

Apart from facilitating antibody evasion, the mutations in the Omicron S protein alter viral entry into host cells. Thus, the Omicron variant infects lung cells, including Calu-3 lung cells, with markedly reduced efficiency as compared to all previously circulating SARS-CoV-2 variants of concern (VOC) and this defect was found to be associated with an increased dependence on the S protein-activating endosomal host-cell protease cathepsin L relative to the cell surface serine protease TMPRSS2^3,8–11^. Similarly, the S protein of the Omicron variant showed a reduced ability to fuse lung cells, a property that is believed to contribute to SARS-CoV-2 pathogenesis^9,11,12^. Thus, the S protein of the Omicron variant might be less adept in facilitating infection and fusion of lung cells and this might account for the reduced ability of the Omicron variant to cause severe disease^8–10^.

**Fig. 1:**
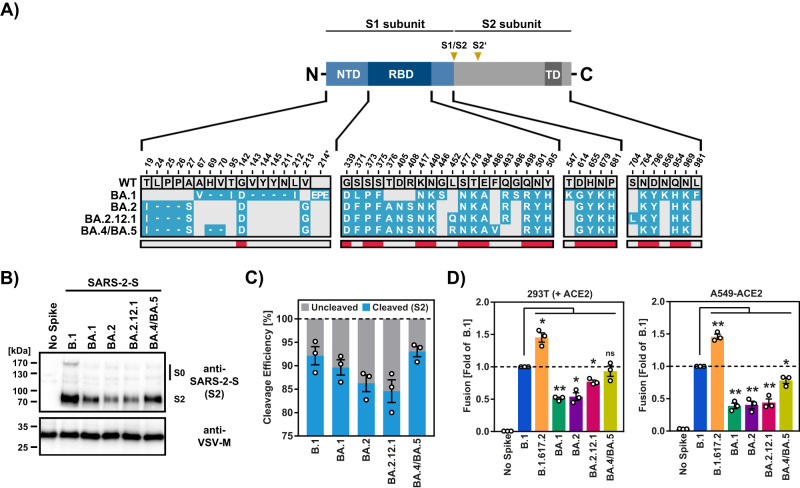
Increased cell–cell fusion driven by the S protein of BA.2.12.1 and BA.4/BA.5. **A** Shared and unique mutations in the S proteins of SARS-CoV-2 Omicron variants BA.1, BA.2, BA.2.12.1, BA.4 and BA.5. Dashes indicate deletions. Gray areas indicate amino acids conserved in certain strains. Red areas indicate amino acids conserved in all strains analyzed. **B** Efficiency of S protein cleavage. Immunoblot analysis of pseudotyped particles containing the indicated S proteins was used to examine S protein particle incorporation and cleavage. S proteins and VSV-M (loading control) were detected by anti-S2 and anti-VSV-M antibodies, respectively. The results were confirmed in two separate experiments. **C** Quantification of S protein cleavage efficiency. Total S protein signals (bands indicating unprocessed [S0] and processed [S2] S protein) for each S protein were set to 100% and the relative proportions of S0 and S2 were determined. The average (mean) data from three biological replicates are shown. Error bars indicate SEM. **D** Spike protein-driven cell–cell fusion. 293T effector cells transiently expressing the indicated S proteins (or no S protein) along with the beta-galactosidase alpha fragment were mixed with either 293T target cells transiently expressing ACE2 and the beta-galactosidase omega fragment, or A549-ACE2 target cells transiently expressing the beta-galactosidase omega fragment. Subsequently, beta-galactosidase substrate was added and luminescence measured. Presented are the average (mean) data ± SEM of three biological replicates, each performed with four technical replicates. For all panels statistical significance was analyzed by two-tailed Student’s *t*-tests with Welch correction (*p* > 0.05, not significant [ns]; *p* ≤ 0.05, *; *p* ≤ 0.01, **; *p* ≤ 0.001, ***), see also Extended Data Table 1. NTD N-terminal domain, RBD receptor binding domain, TD transmembrane domain.

The Omicron subvariants BA.1 and BA.2 dominated the COVID-19 pandemic in the first half of 2022. However, BA.1 circulation decreased rapidly while BA.2 subvariants became prevalent, among them BA.2.12.1, which was responsible for many cases in North America, South America and Europe from 03/2022 to 07/2022, and BA.4 and BA.5^13^. While BA.4 was responsible for a subset of the cases in Europe from May to September, the subvariant BA.5 and its descendants dominated the pandemic in autumn of 2022^14^. The S proteins of BA.4/BA.5 are identical on the amino acid levels and compared to BA.2.12.1 harbor shared and unique mutations in functionally relevant domains (Fig. 1A), including the receptor binding domain (RBD), which facilitates engagement of the cellular receptor ACE2. However, it is currently unknown whether these variants, like BA.1 and BA.2, exhibit inefficient lung cell entry.

Here, we show that in cell culture BA.5 infects lung cells with similar efficiency as B.1, a virus which circulated early in the COVID-19 pandemic. Furthermore, we demonstrate that BA.5 unlike BA.1 efficiently infects the nasal cavity in ferrets and lung tissue in mice, suggesting that BA.5 has an elevated capacity to spread in the respiratory tract and potentially to cause severe disease.

## Results

### BA.4/BA.5 spike protein efficiently fuses cells

We first asked whether BA.2.12.1 and BA.4/BA.5 S proteins exhibit altered cleavage at the S1/S2 site, which occurs in transfected and infected cells and is mediated by the host-cell protease furin^15,16^. The S protein of SARS-CoV-2 variant B.1 exhibits an amino acid sequence identical to that of the Wuhan-Hu-1 S protein but contains mutation D614G and was used as control. We found that all S proteins studied were readily detectable in particle preparations, although levels of BA.2 and BA.2.12.1S protein were reduced relative to B.1 S protein. Cleavage of BA.1 and particularly BA.2 and BA.2.12.1 S proteins was less efficient as compared to B.1 S protein (Fig. 1B, C) while BA.4/BA.5 S protein was cleaved with similar efficiency as B.1 S (Fig. 1B, C). Although an impact of S protein expression levels on our analysis of S protein cleavage cannot be excluded, these results suggest that the BA.4/BA.5 S protein exhibits increased cleavability relative to its counterparts in previously circulating Omicron subvariants. Augmented S protein cleavage was found to be associated with increased cell–cell fusion in the context of the Delta variant (B.1.617.2)^17^ while cell–cell fusion of the Omicron subvariants BA.1 and BA.2 was reported to be reduced^9,11,12^. Therefore, we next analyzed the capacity of BA.2.12.1 and BA.4/BA.5 S proteins to drive cell–cell fusion. Employing 293T effector cells transfected to express S protein and either 293T or A549-ACE2 target cells, we confirmed that cell–cell fusion driven by the S protein of variant B.1.617.2 was increased as compared to B.1 spike while cell–cell fusion driven by the S proteins of BA.1 and BA.2 was reduced (Fig. 1D). Notably, the S protein of BA.2.12.1 showed an intermediate phenotype with 293T-ACE2 but not A549-ACE2 cells, potentially due to differences in ACE2 expression levels, while the S protein of BA.4/BA.5 drove cell–cell fusion with similar efficiency as B.1 S protein (Fig. 1D). These results indicate that the SARS-CoV-2 Omicron subvariants BA.4 and BA.5 might exhibit increased S protein cleavage at the S1/S2 site and ability to fuse lung cells relative to previously circulating Omicron subvariants BA.1 and BA.2.

### Robust ACE2 binding of BA.4/BA.5 spike protein

**Fig. 2:**
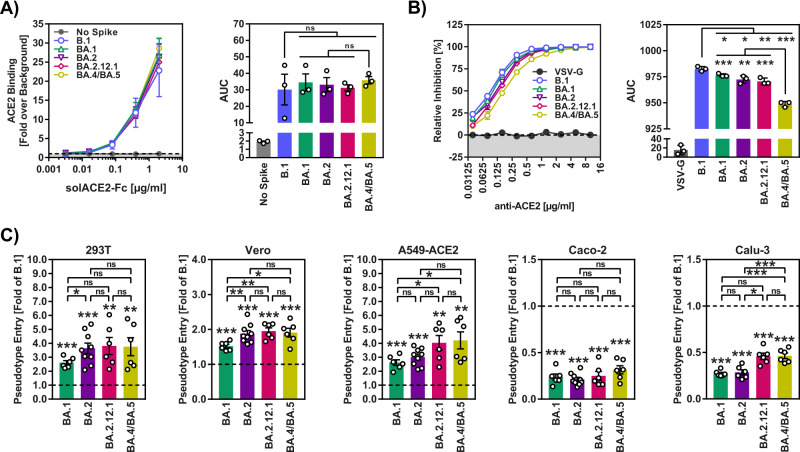
Increased virus-cell fusion driven by the S protein of BA.2.12.1 and BA.4/BA.5. **A** Efficiency of ACE2 binding. Left: 293T cells transiently expressing the indicated S proteins (or no S protein) were incubated with the indicated concentrations of soluble ACE2 harboring a C-terminal Fc-tag (derived from human immunoglobulin G; solACE2-Fc) and then incubated with an AlexaFluor-488-coupled secondary antibody. Subsequently, ACE2 binding was analyzed by flow cytometry and normalized against the assay background (signals for samples without soluble ACE2, set as 1). Right: Area under the curve (AUC) data for ACE2 binding. Both panels show average (mean) data ± SEM from three biological replicates (each with single samples). Please also see Supplementary Fig. 1. **B** Impact of ACE2 blockade on S protein-driven cell entry. Left: Vero cells were preincubated with different concentrations of anti-ACE2 antibody and subsequently inoculated with pseudoviruses bearing the indicated S proteins or VSV-G (or no S protein). Cell entry was assessed by measuring the activity of pseudovirus-encoded firefly luciferase in cell lysates at 16–18 h after inoculation and normalized against samples that were not exposed to anti-ACE2 antibody (set as 0% inhibition). Right: AUC data for ACE2 blockade. Both panels show average (mean) data ± SEM from three biological replicates (each with four technical replicates). **C** Cell entry mediated by S proteins. Cell entry was assessed by measuring the activity of pseudovirus-encoded firefly luciferase in cell lysates at 16–18 h after inoculation of cells with particles containing the indicated S proteins (or no S protein). The average (mean) data ± SEM from 6 to 12 biological replicates (each with four technical replicates) are presented, with entry standardized against B.1 (set as 1). Please also see Supplementary Fig. 2. For all panels statistical significance was analyzed by two-tailed Student’s *t*-tests with Welch correction (*p* > 0.05, not significant [ns]; *p* ≤ 0.05, *; *p* ≤ 0.01, **; *p* ≤ 0.001, ***), see also Extended Data Table 2. AUC area under the curve.

In order to exclude that phenotypes observed in the cell–cell and virus-cell (below) fusion assay might reflect alterations in binding to the cellular receptor ACE2, we next investigated whether the S proteins of BA.2.12.1 and BA.4/BA.5 bound to ACE2 with different efficiency as compared to BA.1 and BA.2 S proteins. For this, we examined binding of ACE2 fused to the Fc portion of human immunoglobulin G to cells transfected to express S proteins, as previously reported^3^. We found that all S proteins analyzed bound to ACE2 with comparable efficiency (Fig. 2A, Supplementary Fig. 1), indicating that differences in cell–cell fusion and virus-cell fusion (see below) were not due to altered ACE2 binding efficiency. Similarly, an anti-ACE2 antibody efficiently blocked Vero cell entry of rhabdoviral reporter particles pseudotyped with all S proteins studied, although inhibition of entry driven by S proteins of Omicron subvariants was slightly less efficient as compared to entry driven by B.1 S protein and blockade of BA.4/BA.5 S protein-mediated entry was least efficient (Fig. 2B, Supplementary Fig. 1).

### Augmented lung cell entry driven by the BA.4/BA.5 spike protein

We next analyzed whether the increased cell–cell fusion driven by BA.2.12.1 and particularly BA.4/BA.5 S proteins was associated with increased cell entry. For this, we employed pseudotyped particles (pp), which mirror key aspects of SARS-CoV-2 cell entry^18^, and cell lines commonly used for SARS-CoV-2 research: 293T (human, kidney), Vero (African green monkey, kidney), A549-ACE2 (human, lung, stably expressing ACE2), Caco-2 (human, colon) and Calu-3 (human, lung). In line with previous reports, particles pseudotyped with BA.1 (BA.1_pp_) or BA.2 (BA.2_pp_) S protein were more efficient at entering 293T, Vero and A549-ACE2 cells, while entry into Caco-2 and Calu-3 cells was reduced as compared to B.1_pp_^3,10^ (Fig. 2C, Supplementary Fig. 2). Further, we found that 293T, Vero, A549-ACE2 and Caco-2 cell entry of BA.2.12.1_pp_ and BA.4/BA.5_pp_ was comparable to that of BA.2_pp_ and, for 293T, Vero and A549-ACE2 cells, was slightly more efficient than that measured for BA.1_pp_ (Fig. 2C). In contrast, Calu-3 cell entry of BA.2.12.1_pp_ and BA.4/BA.5_pp_ was significantly more efficient (on average 1.7-fold increase) than that measured for BA.1_pp_ and BA.2_pp_ (Fig. 2C). Thus, the S proteins of BA.2.12.1 and BA.4/BA.5 have acquired the ability to mediate lung cell entry with higher efficiency as compared to their counterparts from the previously circulating Omicron subvariants BA.1 and BA.2.

### No apparent differences in protease choice between the spike proteins of BA.1, BA.2 and BA.4/BA.5

**Fig. 3:**
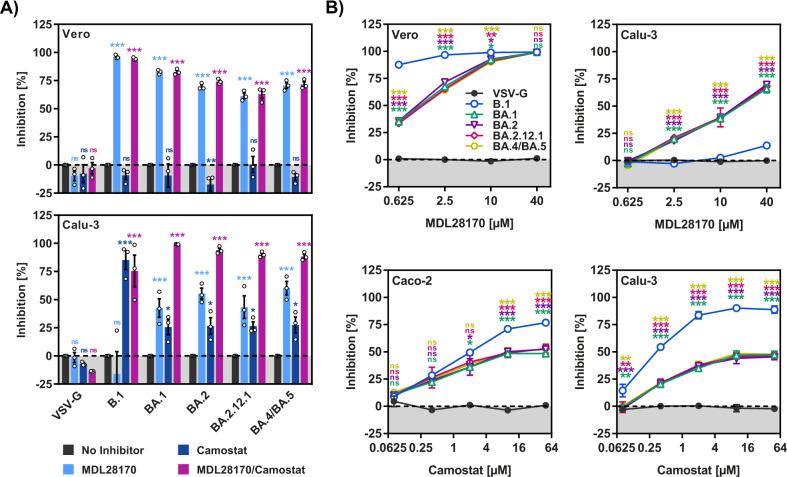
Protease preference of BA.5 S protein is comparable to that of BA.1 and BA.2. **A** Inhibition of S protein-mediated cell entry by protease inhibitors. Target cells were preincubated (1 h, 37 °C) in the presence of no inhibitor, MDL28170 (20 µM), camostat (20 µM) or a combination of MDL28170 and camostat (20 µM each) before pseudoviruses were added. Inhibition of cell entry was analyzed. The average (mean) data ± SEM from three biological replicates (each with four technical replicates) are presented, with entry standardized against no inhibitor-treated cells (set as 0% inhibition). **B** Concentration-dependent inhibition of S protein-mediated cell entry by MDL28170 and camostat. Target cells were preincubated (1 h, 37 °C) in the presence of no inhibitor or different concentrations of MDL28170 or camostat before pseudoviruses were added. Inhibition of cell entry was analyzed. The average (mean) data ± SEM from three biological replicates (each with four technical replicates) are presented, with entry standardized against no inhibitor-treated cells (set as 0% inhibition). Statistical analyses: two-way analysis of variance with Dunnett’s post-hoc test was used to determine statistical significance compared to no inhibitor-treated cells (*p* > 0.05, not significant [ns]; *p* ≤ 0.05, *; *p* ≤ 0.01, **; *p* ≤ 0.001, ***), see also Extended Data Table 3.

The augmented entry of BA.2.12.1_pp_ and BA.4/BA.5_pp_ into Calu-3 cells (relative to BA.1_pp_ and BA.2_pp_) might have been associated with a change in the relative dependence on the host-cell proteases cathepsin L and TMPRSS2 for S protein activation. However, inhibition experiments with protease inhibitors showed that this was not the case: BA.1_pp_, BA.2_pp_, BA.2.12.1_pp_ and BA.4/BA.5_pp_ exhibited comparable sensitivity to the cathepsin L inhibitor MDL28170 and similar results were obtained with the TMPRSS2 inhibitor camostat (Fig. 3A, B). Further, when both TMPRSS2 and cathepsin L were available for entry (Calu-3 and Caco-2 cells) pseudoparticles bearing Omicron S proteins were more sensitive to MDL28170 and less sensitive to camostat as compared to B.1_pp_ (Fig. 3A, B), reflecting the previously noted preference of Omicron subvariants for cathepsin L. Collectively, these results indicate that increased lung cell entry of BA.2.12.1_pp_ and BA.4/BA.5_pp_ (relative to BA.1_pp_ and BA.2_pp_) was not due to changes in protease preference.

### Deletion of H69 and V70 is required for enhanced lung cell entry driven by BA.4/BA.5 spike protein

**Fig. 4:**
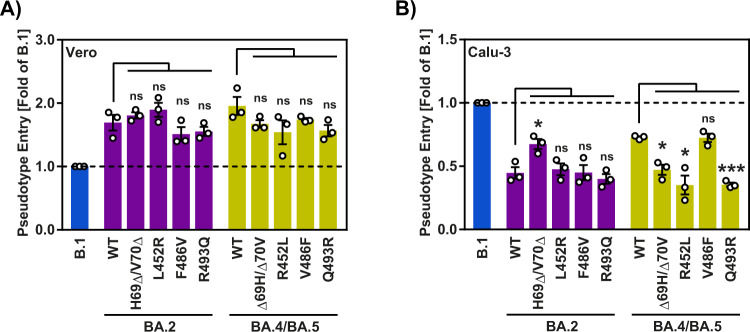
Deletion of H69 and V70 is required for robust lung cell entry driven by  BA.4/BA.5 S protein. Cell entry mediated by mutated BA.2 and BA.4/BA.5 S proteins. Vero (**A**) and Calu-3 (**B**) cell entry was assessed by measuring the activity of virus-encoded firefly luciferase in cell lysates at 16–18 h after inoculation with particles containing the indicated S proteins. The average (mean) data ± SEM from three biological replicates (each with four technical replicates) are presented, with entry standardized against B.1 (set as 1). Statistical significance was analyzed by two-tailed Student’s *t*-tests with Welch correction (*p* > 0.05, not significant [ns]; *p* ≤ 0.05, *; *p* ≤ 0.01, **; *p* ≤ 0.001, ***), please see also Extended Data Table 4 and Supplementary Fig. 2.

In order to determine which residues in the S protein are responsible for the increased lung cell entry of BA.4/BA.5_pp_, we subjected the S proteins of BA.2 and BA.4/BA.5 to mutagenic analysis, focusing on the BA.4/BA.5 S protein-specific amino acid exchanges H69Δ/V70Δ, L452R, F486V and R493Q. None of these mutations significantly impacted cell–cell fusion (Supplementary Fig. 3) and Vero cell entry of BA.2_pp_ and BA.4/BA.5_pp_, respectively (Fig. 4A). L452R and R493Q were important for Calu-3 cell entry of BA.4/BA.5_pp_ but the reverse exchanges failed to increase Calu-3 cells entry of BA.2_pp_ (Fig. 4B). Finally, H69Δ/V70Δ was required for enhanced BA.4/BA.5_pp_ entry into Calu-3 cells and the reverse exchanges increased Calu-3 cell entry of BA.2_pp_ (Fig. 4B), indicating that deletion of H69 and V70 is required for the enhanced lung cell entry of BA.4/BA.5.

### BA.5 infects Calu-3 lung cells with high efficiency

**Fig. 5:**
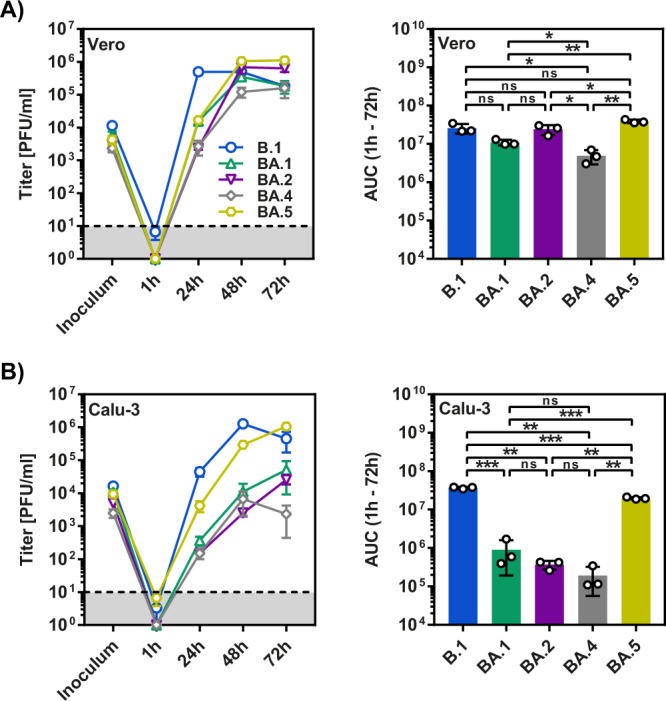
Efficient replication of authentic SARS-CoV-2 BA.5 in Calu-3 lung cells. Vero (**A**) and Calu-3 (**B**) cells were inoculated with authentic SARS-CoV-2 lineages B.1, BA.1, BA.2, BA.4 and BA.5 at an MOI of 0.01, and viral titers in supernatants at the indicated time points were quantified by plaque assay. Left panels show mean titers from three biological replicates (performed with two technical replicates), while right panels represent AUC data (for the time interval between 1 and 72 h). Error bars indicate the standard deviation (growth curves) or the SEM (AUC data). Statistical significance was analyzed by two-tailed Student’s *t*-tests with Welch correction (*p* > 0.05, not significant [ns]; *p* ≤ 0.05, *; *p* ≤ 0.01, **; *p* ≤ 0.001, ***), see also Extended Data Table 5. AUC area under the curve, PFU plaque forming unit.

We next determined whether increased lung cell entry of BA.4/BA.5_pp_ translated into increased lung cell infection by authentic BA.4 and BA.5. For this, we compared infection of Vero and Calu-3 cells with BA.1, BA.2, BA.4 and BA.5 (Fig. 5A). All viruses replicated robustly in Vero cells, although replication of B.1 was most efficient and replication of BA.1 and BA.4 was slightly reduced as compared to the other variants tested (Fig. 5A). Further, Calu-3 cell infection by BA.1, BA.2 and, surprisingly, also BA.4 was roughly a 100-fold less efficient as compared to B.1 (Fig. 5B). Finally, BA.5 replicated in Calu-3 cells with almost the same efficiency as B.1 (Fig. 5B), indicating that BA.5 had acquired the ability to efficiently infect lung cells.

### BA.5 efficiently replicates in the lungs of mice

**Fig. 6:**
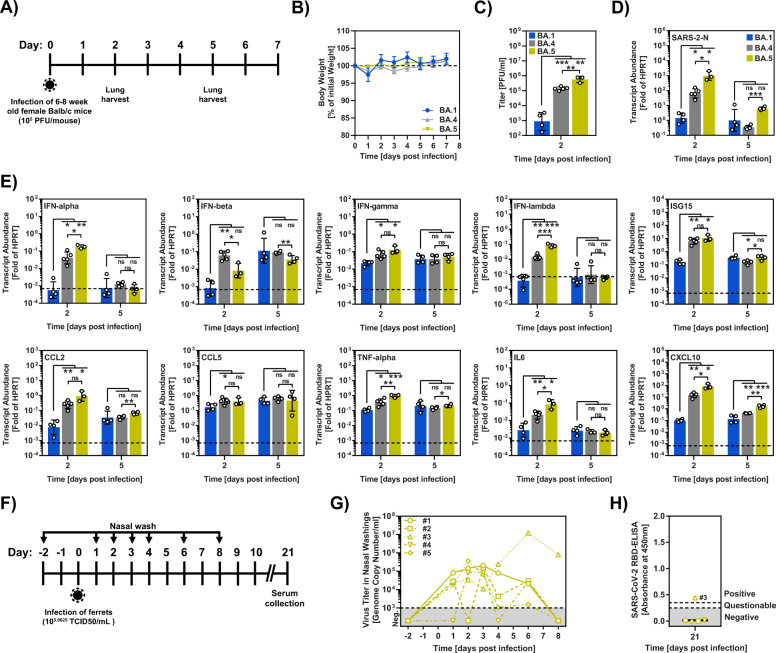
Replication of BA.5 in nasal cavity and lungs. **A** Experimental timeline of mouse infection study with Omicron variants. **B** Percent of initial weight of mice infected with 10^5^ PFU of the indicated variant. Infected mice were monitored and weighed daily for up to day 7 post infection. The dotted line indicates 100% of weight. The average of five animals per time point is shown, error bars indicate SEM. **C** Infectious virus titers in the lungs of mice infected with 10^5^ PFU Omicron variants, as described for **B**, were determined by plaque assay in Vero-hACE2-TMPRSS2 cells. Geometric mean viral titers for three to five mice are shown, error bars indicate geometric mean standard deviation. **D** N gene transcripts levels in the lungs of mice infected with 10^5^ PFU Omicron variants were determined at the indicated time point by quantitative PCR. Presented are the geometric mean transcript levels for three to five animals (each sample was measured in technical duplicates), error bars indicate geometric mean standard deviation. **E** Levels of selected cytokines/chemokines in the lungs of mice infected with 10^5^ PFU Omicron variants were determined at the indicated time point by quantitative PCR. Presented is the geometric mean transcript abundance for three to five animals (each sample was measured in technical duplicates), error bars indicate geometric mean standard deviation. In **C**–**E**, four animals (BA.1 group), five animals (BA.4 group) and three animals (BA.5 group) were analyzed at 2 days post infection while groups of four animals each were analyzed at 5 days post infection. Statistical significance for **C**–**E** was determined by two-tailed Student’s *t*-test (*p* > 0.05, not significant [ns]; *p* ≤ 0.05, *; *p* ≤ 0.01, **; *p* ≤ 0.001, ***), see also Extended Data Table 6. **F** Timeline of the ferret infection study with SARS-CoV-2 Omicron BA.5. **G** Nasal washings of BA.5-inoculated ferrets. Nasal washings were performed for 4 consecutive days and subsequently every 2 days until 8 dpi. Viral genome copies were measured by including a standard of a known SARS-CoV-2 RNA concentration. **H** Serological analysis of individual ferret sera from 21 dpi via a SARS-CoV-2 Wuhan-strain-based RBD-ELISA. Values > 0.3 = positive and <0.3 = negative.

We next examined whether the robust Calu-3 lung cell infection of BA.5 observed in cell culture translated into efficient replication of BA.5 in the lungs of mice. For this, we intranasally infected Balb/c mice with BA.5 and studied body weight, viral replication and induction of cytokine expression (Fig. 6A). BA.1 and BA.4 were examined in parallel. None of the mice had to be euthanized due to any severe clinical signs after infection and no decrease in body weight was observed upon infection with all variants tested (Fig. 6B). However, analysis of infectious units and viral genome copies showed that BA.5 replicated in lungs about 1000-fold more efficiently than BA.1 (Fig. 6C, D). Somewhat surprisingly, BA.4 also replicated robustly in lung tissue, although roughly tenfold less efficiently than BA.5 (Fig. 6C, D). Finally, BA.5 induced expression of certain cytokines, including IL-6, with higher efficiency than BA.1 while BA.4 showed an intermediate phenotype (Fig. 6E). Collectively, these results indicate that BA.5 unlike the previously circulating Omicron subvariants BA.1 and BA.2 can efficiently replicate in lung tissue.

### BA.5 replicates in the nasal epithelium of ferrets

Ferrets are naturally susceptible to SARS-CoV-2 infection, allow for virus amplification in the nasal epithelium and allow for contact transmission of the virus^19,20^. We had previously found that ferret infection by the Omicron subvariant BA.1 was abortive, and all inoculated animals did not seroconvert^21^. Therefore, we investigated whether inoculation of ferrets with BA.5 resulted in virus replication and disease (Fig. 6F). Virus replication was detected in the nasal cavity of all inoculated animals (Fig. 6G) and one animal seroconverted (Fig. 6H), demonstrating that BA.5 acquired increased replicative capacity in the upper respiratory tract of ferrets as compared to BA.1.

## Discussion

Our results show that, unlike the previously circulating Omicron subvariants BA.1 and BA.2, the subvariant BA.5 efficiently enters human lung cells and replicates in the upper (ferrets) and lower (mice) respiratory tract. These results suggest that BA.5 might have increased capacity to cause severe disease as compared to previously circulating Omicron subvariants.

The fusion of SARS-CoV-2 infected cells with neighboring uninfected cells is driven by the S protein and results in the formation of syncytia, which might contribute to COVID-19 pathogenesis^22,23^. The Omicron subvariants BA.1, BA.2 and BA.3 are less well able to fuse cells as compared to previously circulating variants, including the Delta variant, and reduced cell–cell fusion might partially account for the reduced pathogenic potential of these Omicron subvariants as compared to previously circulating variants of concern^9,11,12,24^. The S proteins of BA.2.12.1 and particularly BA.4/BA.5 showed increased cell–cell fusion as compared to the BA.1 and BA.2 S proteins and it will be interesting to determine whether syncytium formation is increased in BA.2.12.1 and BA.4/BA.5 patients. Further, the efficiency of syncytium formation has been associated with the efficiency of S protein cleavage^17,25^. It is therefore noteworthy that the presence of an additional arginine residue in the S1/S2 cleavage site that is found in certain BA.5 subvariants (exchange H681R) tended to have a more prominent effect on B.1 (exchange P681R) than BA.4/BA.5 S protein cleavage and virus-cell fusion (Supplementary Fig. 4).

The Omicron subvariants BA.1 and BA.2 fail to efficiently infect lung cells, potentially due to inefficient usage of the protease TMPRSS2, and this phenotype might partially account for the reduced capacity of these variant to cause severe disease^3,9–12^. The present study shows that BA.2.12.1_pp_ and BA.4/BA.5_pp_ entered lung cells more robustly and this phenotype was dependent on H69Δ/V70Δ, mutations that were previously linked to increased infectivity^26,27^, but was not associated with augmented TMPRSS2 usage. Thus, sensitivity of BA.2.12.1_pp_ and BA.4/BA.5_pp_ to the TMPRSS2 inhibitor camostat was unchanged relative to BA.1_pp_ and BA.2_pp_ and entry of BA.2.12.1_pp_ and BA.4/BA.5_pp_ into TMPRSS2^+^ Caco-2 cells was not increased as compared to BA.1_pp_ and BA.2_pp_. In contrast, a study examining entry of patient-derived SARS-CoV-2 into 293T cells engineered to express ACE2 and TMPRSS2 reported increased TMPRSS2 usage of BA.5 over BA.1 and BA.2^28^, potentially due to difference in receptor and protease expression levels compared to Calu-3 and Caco-2 cells examined in the present study. We speculate that augmented Calu-3 lung entry might be associated with use of certain attachment-promoting factors or evasion of restriction factors of the innate immune system. Indeed, initial experiments with amphotericin B, which rescues SARS-CoV-2 infection from blockade by the endo-/lysosomal restriction factors IFITM2/IFITM3^29–31^, suggested that BA.4/BA.5 might be less susceptible to inhibition by these factors as compared to BA.1 and BA.2 (Supplementary Fig. 5), although this effect was not statistically significant.

The robust entry of BA.4/BA.5_pp_ into Calu-3 cells correlated with efficient BA.5 infection of these cells and with BA.5 spread in the nasal cavity of ferrets and the lung of Balb/c mice. This correlation was not observed for BA.4. Despite robust Calu-3 cell entry of BA.4/BA.5_pp_, BA.4 infection of Calu-3 cells was low (i.e. similar to that measured for BA.1). Nevertheless, the virus replicated robustly in mouse lungs. The reason for this discrepancy is at present unknown but one could speculate that a genetic determinant other than the S gene might limit viral spread in human but not mouse lung cells.

Our study reveals that BA.5 has acquired increased capacity to infect lung cells. However, it remains to be determined whether this translates into increased virulence. Recent reports provide initial insights. Two studies examining BA.5 infection in hamster and mouse models detected no apparent differences in lung infection and pathogenicity between BA.2 and BA.5, although competition experiments indicated greater replicative fitness of BA.5 relative to BA.2^32,33^. In contrast, two separate studies demonstrated augmented lung infection and higher pathogenicity of BA.5 as compared to BA.2 in hamster models, with BA.5 but not BA.2 infected animals losing weight and showing extensive lung damage^34,35^. Regarding BA.5 infection of humans, two studies examining patients in South Africa suggested that BA.5 infection was not associated with more severe disease as compared to infection with previously circulating Omicron subvariants^36,37^. It should be noted, however, that the South African population is relatively young and contains a high percentage of previously infected or vaccinated individuals. As a consequence, the impact of BA.5 on the health of older populations with lower levels of preexisting immunity might be more severe. Indeed, studies examining patients in Denmark^38^ and Canada^39^ reported that risk of hospitalization was increased for BA.5 as compared to BA.1 (Canada) and BA.2 (Denmark) infected patients, respectively.

In sum, the present study and recent reports show augmented lung infection and possibly pathogenicity of BA.5 relative to previously circulating Omicron subvariants, indicating that SARS-CoV-2 evolution might, at least in the short term, not result in attenuation.

## Methods

### Cell culture

293T (human, female, kidney; ACC-635, DSMZ; RRID: CVCL_0063), A549 cells (human, lung; CRM-CCL-185, ATCC, RRID:CVCL_0023; kindly provided by Georg Herrler), Vero (African green monkey kidney, female, kidney; CRL-1586, ATCC; RRID: CVCL_0574, kindly provided by Andrea Maisner), Vero-hACE2-TMPRSS2 (African green monkey kidney, female, kidney; BEI resources, NR-54970) and Huh-7 (human, male, liver; JCRB Cat# JCRB0403; RRID: CVCL 0336, kindly provided by Thomas Pietschmann) cells were cultured in Dulbecco’s modified Eagle medium (DMEM, PAN-Biotech). Calu-3 (human, male, lung; HTB-55, ATCC; RRID: CVCL_0609, kindly provided by Stephan Ludwig) and Caco-2 cells (human, male, colon; HTB-37, ATCC, RRID: CVCL_0025; kindly provided by Georg Herrler) were cultured in minimum essential medium (MEM, GIBCO). A549-ACE2 cells^40^ were derived from parental A549 cells and were cultured in DMEM/F-12 Medium with Nutrient Mix (ThermoFisher Scientific) and supplemented with 1 μg/ml puromycin. All media were supplemented with 10% fetal bovine serum (FBS, Biochrom), 100 U/ml penicillin and 0.1 mg/ml streptomycin (pen/strep) (PAN-Biotech). Growth media for Caco-2 and Calu-3 cells were further supplemented with 1× non-essential amino acid solution (from 100× stock, PAA) and 1 mM sodium pyruvate (PAN-Biotech). Cell lines were validated using STR-typing, amplification and sequencing of a cytochrome c oxidase gene fragment, microscopic examination, and/or growth characteristics. Furthermore, mycoplasma contamination was routinely tested. All cell lines were incubated at 37 °C in a humidified atmosphere containing 5% CO_2_.

### Plasmids

Plasmids pCAGGS-DsRed^41^, pCAGGS-VSV-G (vesicular stomatitis virus glycoprotein)^42^, pCG1-SARS-CoV-2 B.1 SΔ18 (codon-optimized, C-terminal truncation of 18 amino acid residues, GISAID Accession ID: EPI_ISL_425259)^40^, pCG1-SARS-CoV-2 BA.1 SΔ18 (GISAID Accession ID: EPI_ISL_6640919)^3^, pCG1-SARS-CoV-2 BA.2 SΔ18 (GISAID Accession ID: EPI_ISL_8738174), as well as plasmids encoding the beta-galactosidase alpha and omega fragment within plasmid pQCXIP^24^ have been previously described. Using five overlapping DNA strings (Thermo Fisher Scientific), BamHI/XbaI-digested pCG1 plasmid, and GeneArt^TM^ Gibson Assembly HiFi Master Mix, Gibson assembly was used to generate expression plasmids for SARS-CoV-2 BA.2.12.1 SΔ18 (GISAID Accession ID: EPI ISL 12028907) and SARS-CoV-2 BA.4/BA.5 SΔ18 (GISAID Accession ID: EPI_ISL_11550739 and EPI_ISL_12029894). The manufacturer’s instructions were followed for preparing the reactions. Point mutations were introduced into the spike gene by overlap-extension PCR. The sequences of the oligonucleotides used for this study are provided in the Extended Data Table 10. Roberto Cattaneo, Mayo Clinic College of Medicine, Rochester, MN, USA, generously provided the pCG1 expression plasmid. A commercial sequencing service was used to verify all PCR-amplified sequences (Microsynth SeqLab). The GISAID (global effort on sharing all influenza data) database (https://www.gisaid.org/) was used to gather S protein sequences and the underlying metadata (collection date, location).

### ACE2 binding

293T cells were seeded in 6-well plates and transfected with expression plasmids for the corresponding SARS-CoV-2 S protein by calcium-phosphate precipitation. As negative control, cells were transfected with an empty plasmid. The medium was changed at 24 h after transfection. Medium was removed at 48 h after transfection, and the cells were resuspended in PBS and transferred to 1.5 ml reaction tubes before being pelleted by centrifugation. All centrifugation procedures were carried out at room temperature for 5 min at 600 × *g*. The supernatant was then aspirated, and the cells were rinsed in PBS containing 1% bovine serum albumin (BSA, PBS-B) and pelleted. The cell pellets were then resuspended in 250 µl PBS-B containing different concentrations of soluble solACE2-Fc (Bio-Techne) and rotated for 60 min at 4 °C using a Rotospin test tube rotator disk (IKA). Cells were pelleted, resuspended in 250 µl PBS-B containing goat anti-Human IgG (H + L) cross-adsorbed secondary antibody, Alexa Fluor™ 488 (1:200, Thermo Fisher Scientific, Catalog # A-11013), and rotated for 60 min at 4 °C. Finally, the cells were washed in PBS-B, fixed for 30 min at room temperature in a 1 % paraformaldehyde solution, washed again, and resuspended in 100 µl PBS-B before being analyzed with an ID7000 Spectral Cell Analyzer (Sony Biotechnology, San Jose, CA, USA). Mean channel fluorescence data were further analyzed using the ID7000 software.

### Immunoblot

To investigate S protein cleavage and particle incorporation, vesicular stomatitis virus (VSV) pseudotypes bearing S proteins (codon-optimized, with a C-terminal truncation of 18 amino acid residues) were concentrated by high-speed centrifugation (13,300 rpm, 90 min, 4 °C) through a sucrose cushion (20 % w/v sucrose in PBS) and lysed in 2× Sample buffer (0.03 M Tris-HCl, 10% glycerol, 2% SDS, 5% beta-mercaptoethanol, 0.2% bromophenol blue, 1 mM EDTA). Proteins were blotted onto nitrocellulose membranes (Hartenstein) after SDS-PAGE and blocked for 30 min in 5% BSA. After blocking, the membranes were incubated overnight at 4 °C with primary antibodies reactive against S2 (SARS-CoV-2 (2019-nCoV) Spike S2 Antibody, Rabbit PAb, antigen affinity purified (1:2000, Biozol, Cat: SIN-40590-T62)) or anti-VSV-M [23H12] antibody (1:1000, Kerafast, Cat: EB0011). Membranes were then treated with anti-rabbit (S2, goat IgG anti-rabbit IgG (H + L)-HRPO (Dianova, Cat: 111-035-003)) or anti-mouse (VSV-M, goat IgG anti-mouse IgG (H + L)-HRPO (Dianova, Cat: 115-035-003)) secondary antibodies coupled with horseradish peroxidase (1:2000). S2 antibody was diluted in 5% BSA and VSV-M antibody in PBS-T containing 5% skim milk, and blots were washed three times with PBS-T for 10 min after each antibody incubation. Immunoblots were incubated with a home-made chemiluminescence solution (0.1 M Tris-HCl [pH 8.6], 250 g/ml luminol, 0.1 mg/ml para-hydroxycoumaric acid, 0.3 percent hydrogen peroxide) analyzed with the ChemoCam imaging system and ChemoStar Professional software (Intas Science Imaging Instruments). The ImageJ software (version 1.53C, https://imagej.nih.gov/ij/) was used to quantify protein bands. Total S protein signals (uncleaved, S0, and cleaved, S2) were normalized against their respective VSV-M signals for the examination of S protein incorporation into VSV particles, and the resulting values were further normalized against the B.1S protein (set as 1). Total S protein signals (uncleaved, S0, and cleaved, S2) were set to 100% for each S protein for quantification of S protein cleavage, and the contribution of S0 and S2 to the overall signal was determined.

### Production of VSV pseudotypes

Vesicular stomatitis virus particles pseudotyped with the SARS-CoV-2 S proteins were produced as described previously^41^. Using the calcium-phosphate method, 293T cells were transfected with plasmids encoding S protein or VSV-G, or an empty plasmid (control). VSV-G-transcomplemented VSV*G(FLuc), a replication-deficient vesicular stomatitis virus (VSV) that lacks the genetic information for its own glycoprotein (VSV-G) and instead codes for two reporter proteins, enhanced green fluorescent protein (eGFP) and firefly luciferase (kindly provided by Gert Zimmer), was inoculated onto cells 30 h after transfection^43^. The inoculum was removed after 1 h of incubation and the cells were rinsed in phosphate-buffered saline (PBS). After that, all cells received DMEM medium with anti-VSV-G antibody (1:1000, culture supernatant from I1-hybridoma cells; ATCC no. CRL-2700) to neutralize residual VSV-G, with the exception of cells expressing VSV-G, which received medium without antibody. The culture supernatant was taken after 16–18 h of incubation, cleared from cellular debris by centrifugation at 4000 × *g* for 10 min, aliquoted, and stored at −80 °C until further use.

### Transduction of target cells

Target cells seeded in 96-well plates were inoculated with equal volumes of pseudotypes, and transduction efficiency was assessed by detecting luciferase activity in cell lysates at 16–18 h after transduction. For this, cells were lysed for 30 min at room temperature in PBS containing 0.5% Triton X-100 (Carl Roth). Subsequently, luciferase substrate (Beetle-Juice, PJK) was added to cell lysates in white 96-well plates and luminescence was measured using a Hidex Sense plate luminometer (Hidex). For experiments investigating the impact of ACE2 blockade on S protein-driven cell entry, Vero cells were incubated for 30 min at 37 °C with twofold serial dilution of anti-ACE2 antibody (recombinant anti-ACE2 neutralizing antibody (Sino Bilogicals, Cat: 10108-MM36)) starting at 10 µg/ml prior to inoculation with pseudotypes.

For experiments addressing the effects of the antifungal amphotericin B (AmphoB) or the protease inhibitors MDL28170 (inhibitor of cathepsin L) and camostat mesylate (camostat, TMPRSS2 inhibitor), target cells were incubated for 1 h in medium containing the respective compound or solvent (AmphoB, water; MDL28170 and camostat, DMSO) prior to inoculation with pseudotypes.

### Quantitative fusion assay

293T effector cells grown to 75% confluency in 12-well plates were cotransfected with expression plasmids for the respective S protein or empty vector (1.5 µg/well) and the beta-galactosidase alpha fragment (0.5 µg/well) using Lipofectamine 2000 (Thermo Fisher Scientific) according to the manufacturer’s instructions. Subsequently, effector cells were washed, resuspended in 500 µl and added to 293T target cells (96-well format, 100 µl/well, four technical replicates) that were transfected with plasmids encoding ACE2 (0.1 µg/well) and the beta-galactosidase omega fragment (0.1 µg/well), or A549-ACE2 target cells (96-well format, 100 µl/well, four technical replicates) that were transfected with plasmid encoding the beta-galactosidase omega fragment (0.1 µg/well). Beta-galactosidase substrate (Gal-Screen, Thermo Fisher Scientific) was added (100 µl/well) after an additional 24 h of incubation, and samples were incubated for 90 min in the dark at room temperature before they were transferred into white 96-well plates and luminescence was measured using a Hidex Sense plate luminometer (Hidex).

### SARS-CoV-2 infection of cell lines

Vero E6 cells were seeded in 6-well plates at 1.5 × 10^5^ cells/well, Calu-3 cells at 3 × 10^5^ cells/well. Cultured cells were infected with early passage virus stocks at an MOI of 0.01 for 1 h at 37 °C. Supernatants were harvested at the indicated time points and virus was quantified by plaque titration on Vero E6 cells using a previously published protocol^44^. Isolates B.1, BA.1 and BA.2 were from the in-house strain collection of Charité. Isolates BA.4 and BA.5 were obtained from the WHO BioHub resource.

### Infection of mice

Female BALB/c mice of 6–8 weeks of age were obtained from Charles River Laboratories. Mice were maintained in the Animal Care Unit at the University of Iowa under standard conditions of dark/light cycle, ambient temperature and humidity (Lighting—12 light:12 dark cycle, Humidity—30–70%, Temperature range—kept in accordance with the Guide for the Care and Use of Laboratory Animals (https://grants.nih.gov/grants/olaw/Guide-for-the-care-and-use-of-Laboratory-animals.pdf) page 44, 20–26 °C for mouse). Mice were randomly assigned to different groups, with numbers per group sufficient to obtain statistical significance.

Mice were anaesthetized with ketamine–xylazine and infected intranasally with 10^5^ PFU of Omicron variants (BA.1: EPI_ISL_7171744; BA.4: NR-56806, BEI; BA.5: NR-58620, BEI) in a total volume of 50 μl DMEM. Animal weight and health were monitored daily. All mouse experiments with SARS-CoV-2 were performed in a biosafety level 3 (BSL3) laboratory at the University of Iowa.

### Quantification of viral titers in infected mice

At the indicated times, mice were euthanized and transcardially perfused with PBS. Lungs were collected and homogenized before clarification by centrifugation and titring. Tissue homogenates were serially diluted in DMEM. Twelve-well plates of Vero-hACE2-TMPRSS2 cells were inoculated at 37 °C in 5% CO_2_ for 1 h and gently rocked every 15 min. After removing the inocula, plates were overlaid with 0.6% agarose containing 2% FBS. After 2 days, overlays were removed and plaques visualized by staining with 0.1% crystal violet. Viral titers were quantified as PFUs per ml tissue.

### Infection of ferrets

Five female ferrets were kindly provided by the Paul-Ehrlich-Institute (PEI, Langen, Germany) and housed in multiple connected cage units. The animals were intranasally inoculated with 200 µl of SARS-CoV-2 Omicron BA.5 (EPI_ISL_12268493.2) at a concentration of 10^5.0625^ TCID_50_/ml (calculated by back-titration of the inoculum). Ferrets were sampled 2 days before inoculation and for 4 consecutive days after inoculation (starting at 1 dpi), as well as every 2 days from five to eight dpi via nasal washings. In addition, body weight was determined. Nasal washings were performed under a short-term isoflurane inhalation anesthesia via administration of 750 µl PBS directly into each nostril and collection of the reflux. Physiological condition of the ferrets was monitored daily by trained animal caretakers or a veterinarian.

### Quantification of viral RNA in infected mice and ferrets

#### Mice

Total RNA was extracted from tissues using TRIzol (Invitrogen) according to the manufacturer’s protocol. Following DNase treatment, 1 μg of total RNA was used as a template for first-strand cDNA using SuperScript IV RT system (Invitrogen). The resulting cDNA was subjected to amplification of selected genes by real-time quantitative PCR using Power SYBR Green PCR Master Mix (Applied Biosystems). Average values from duplicates of each gene were used to calculate the relative abundance of transcripts normalized to *HPRT* and presented as 2^−ΔCT^. The primers used for cytokine and chemokines were reported previously^45^. For detection of viral genomes, the following primers were used to amplify transcripts for the N protein: 2019-nCoV_N1-F: 5′-GACCCCAAAATCAGCGAAAT-3′; 2019-nCoV_N1-R: 5′-TCTGGTTACTGCCAGTTGAATCTG-3′.

#### Ferrets

One hundred microliters of the collected nasal washes were used for nucleic acid extraction with the NucleoMag Vet kit (Macherey Nagel). Viral genomes were detected and quantified by quantitative real-time polymerase chain reaction (real-time RT-qPCR). Target sequence for the specific amplification was the viral RNA-dependent RNA polymerase gene (WHO. Coronavirus disease (COVID-19) technical guidance: Laboratory testing for 2019-nCoV in humans. Online available: https://www.whoint/emergencies/diseases/novel-coronavirus-2019/technical-guidance/laboratory-guidance). In order to calculate viral genome copy numbers per ml, a standard dilution series with a known copy number concentration - determined by digital droplet PCR - was carried along in each PCR-run.

### Serological analysis of infected ferrets

Serum samples of individual ferrets were analyzed with a multispecies ELISA for sero-reactivity against the Wuhan-strain based SARS-CoV-2 RBD-domain^46^. Immunofluorescence analysis (IFA) was performed by infecting Vero 76 cells (CCLV-RIE-0228, Collection of Cell Lines in Veterinary Medicine) with 10^2,5^ TCID_50_/ml SARS-CoV-2 Omicron BA.5 for 48 h. Subsequently, cells were fixed with 4% PFA (Sigma Aldrich) and permeabilized with 0.1% Triton X-100 (Sigma Aldrich). Cells were incubated with 50 µl of 1:100 or 1:500 prediluted sera of the respective animals for 1 h and subsequently incubated with 50 µl of an α-ferret IgG FITC-conjugated secondary antibody (1:250, Bethyl, A140-108F) for 1 h.

### Statistics

Microsoft Excel (as part of the Microsoft Office software package, version 2019, Microsoft Corporation) and GraphPad Prism 8 version 8.4.3 (GraphPad Software) were used to analyze the data. The tests used to determine statistical significance are indicated in the figure legends.

Only *p* values of 0.05 or less were considered statistically significant (*p* > 0.05, not significant, ns; *p* ≤ 0.05, *; *p* ≤ 0.01, **; *p* ≤ 0.001, ***).

### Ethics committee approval

All mouse studies were approved by the University of Iowa Animal Care and Use Committee and meet stipulations of the Guide for the Care and Use of Laboratory Animals. The ferret infection study was evaluated by the responsible ethics committee of the State Office of Agriculture, Food Safety, and Fishery in Mecklenburg–Western Pomerania (LALLF M-V) and gained governmental approval under the registration number LVL MV TSD/7221.3-2-005/21.

### Reporting summary

Further information on research design is available in the Nature Portfolio Reporting Summary linked to this article.

## Supplementary information

Supplementary Information File

Reporting Summary

## Data Availability

The sequences of SARS-CoV-2 spike proteins were obtained from GISAID database (https://gisaid.org/). All unprocessed data generated in this study are provided in the Supplementary Information. Any additional information required to reanalyze the data reported in this paper is available on request. Source data are provided with this paper.

## Source data

Source Data File
