# Supplementary material for: Omicron subvariant BA.5 efficiently infects lung cells
Source: Nat Commun. 2023 Jun 13;14:3500. doi: 10.1038/s41467-023-39147-4 (PMC10262933; doi:10.1038/s41467-023-39147-4)
Supplement: Supplementary file 1 — Supplementary Information File [file 41467_2023_39147_MOESM1_ESM.pdf]

Supplement to

Omicron subvariant BA.5 efficiently infects lung cells

Markus Hoffmann[1,2,6], Lok-Yin Roy Wong[3,6], Prerna Arora[1,2,6], Lu Zhang[1,2], Cheila Rocha[1,2], Abby Odle[3], Inga Nehlmeier[1], Amy Kempf[1,2], Anja Richter[4], Nico Joel Halwe[5], Jacob Schön[5], Lorenz Ulrich[5], Donata Hoffmann[5], Martin Beer[5], Christian Drosten[4], Stanley Perlman[3], Stefan Pöhlmann[1,2,7]

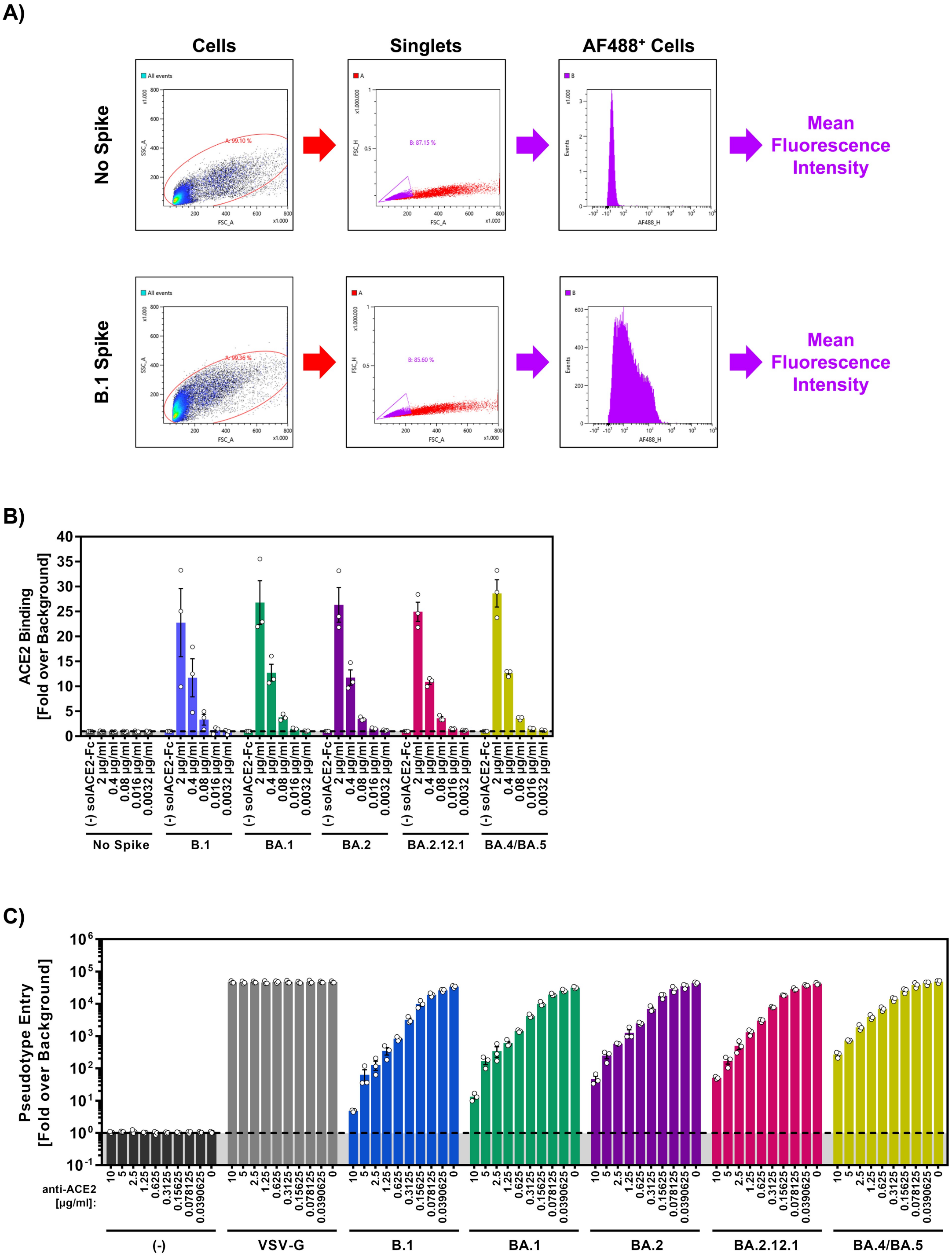

Suppl. Fig. 1 | ACE2 interactions of the S proteins of BA.2.12.1 and BA.4/BA.5 (related to Figure 2).

a Gating strategy for flow cytometry (related to Figure 2a). **b** Bar graphs for the data on the efficiency of ACE2 binding by the indicated S proteins (Figure 2a). Individual experiments are indicated by open circles. Average (mean) data ± SEM from three biological replicates (each with single samples) are shown. **c** Bar graphs for the data on the inhibition of S protein- or VSV-G-driven cell entry by an ACE2-blocking anti-ACE2 antibody (Figure 2b). Individual experiments are indicated by open circles. Average (mean) data ± SEM from three biological replicates (each with four technical replicates) are shown.

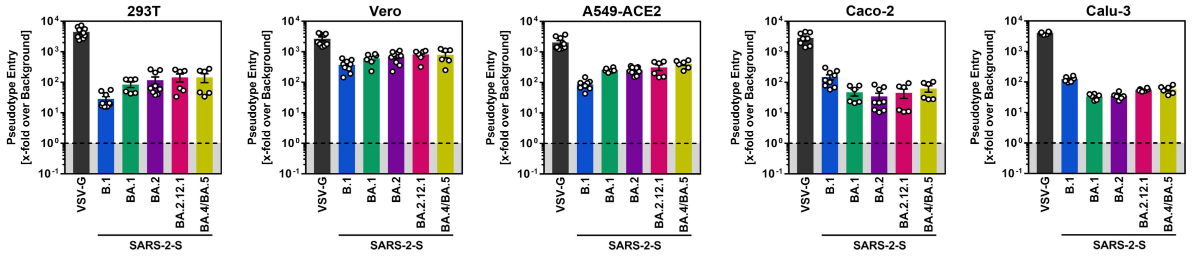

Suppl. Fig. 2 | Cell entry driven by S proteins of BA.2.12.1 and BA.4/BA.5 (related to Figure 2).

Pseudotype entry data (Fig. 2c) normalized against the assay background (cells inoculated with pseudoviruses bearing no viral glycoprotein, set as 1). Further, data for pseudoviruses bearing VSV-G are included. The average (mean) data ± SEM from six to twelve biological replicates (each with four technical replicates) are presented. Error bars indicate the standard error of the mean.

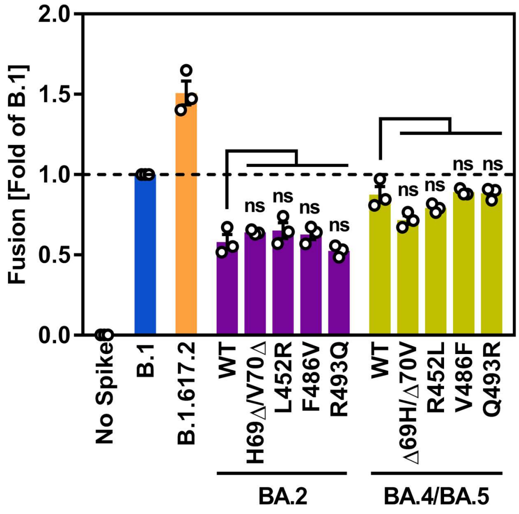

Suppl. Fig. 3 | Cell-cell fusion driven by mutated BA.2 and BA.5 S proteins (related to Fig. 4).

293T effector cells transiently coexpressing the indicated S proteins (or no S protein) along with the beta-galactosidase alpha fragment were mixed with 293T target cells transiently coexpressing ACE2 and the beta galactosidase omega fragment. Subsequently, beta-galactosidase substrate was added and luminescence measured. Presented are the average (mean) data of three biological replicates, each performed with four technical replicates. Error bars indicate SEM. Statistical significance was analyzed by two-tailed Student's t-tests with Welch correction ($p > 0.05$, not significant [ns]; $p \leq 0.05$, *; $p \leq 0.01$, **; $p \leq 0.001$, ***), see also Extended Data Table 7.

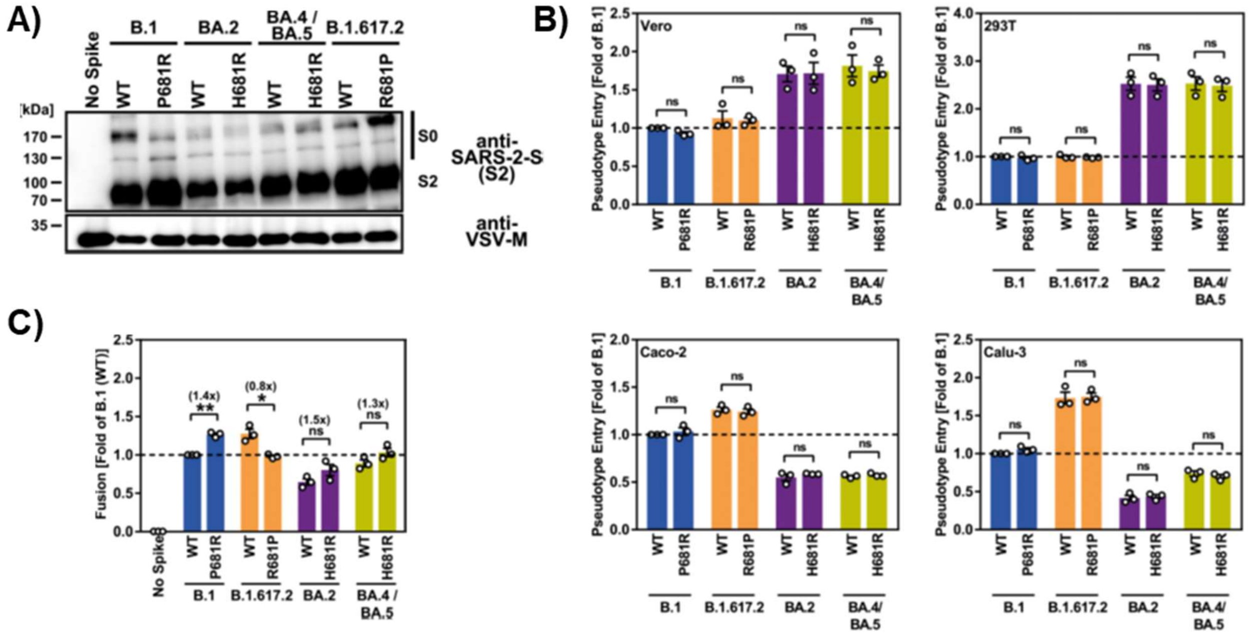

Suppl. Fig. 4 | Impact of mutation H681R on BA.2 and BA.5 S protein cleavage, cell entry and cell-cell fusion (related to the main text, discussion section).

a Immunoblot analysis of pseudotyped particles harboring the indicated S proteins was used to examine S protein particle incorporation and cleavage. S proteins and VSV-M (loading control) were detected by anti-S2 and anti-VSV-M antibodies in combination with peroxidase-conjugated anti-rabbit (S2) and anti-mouse (VSV-M) secondary antibodies, respectively. The findings of a single experiment are presented and were confirmed in two separate experiments. **b** Cell entry mediated by S proteins. Cell entry was assessed by measuring the activity of virus-encoded firefly luciferase in cell lysates at 16-18 hours after inoculation with particles containing the respective S proteins (or no S protein). The average (mean) data from three biological replicates (each with four technical replicates) are presented, with entry standardized against B.1 (set as 1). The SEM is represented by error bars. Statistical significance was analyzed by two-tailed Student's t-tests with Welch correction ($p > 0.05$, not significant [ns]; $p \leq 0.05$, *; $p \leq 0.01$, **; $p \leq 0.001$, ***). **c** S protein driven cell-cell fusion. 293T effector cells transiently coexpressing the indicated S proteins (or no S protein) along with the beta-galactosidase alpha fragment were mixed with 293T target cells

transiently expressing ACE2 and the beta galactosidase omega fragment. Subsequently, beta-galactosidase substrate was added and luminescence measured. Presented are the average (mean) data of three biological replicates, each performed with four technical replicates. For all panels, error bars indicate SEM and statistical significance was analyzed by two-tailed Student's t-tests with Welch correction ($p > 0.05$, not significant [ns]; $p \leq 0.05$, *; $p \leq 0.01$, **; $p \leq 0.001$, ***), see also Extended Data Table 8.

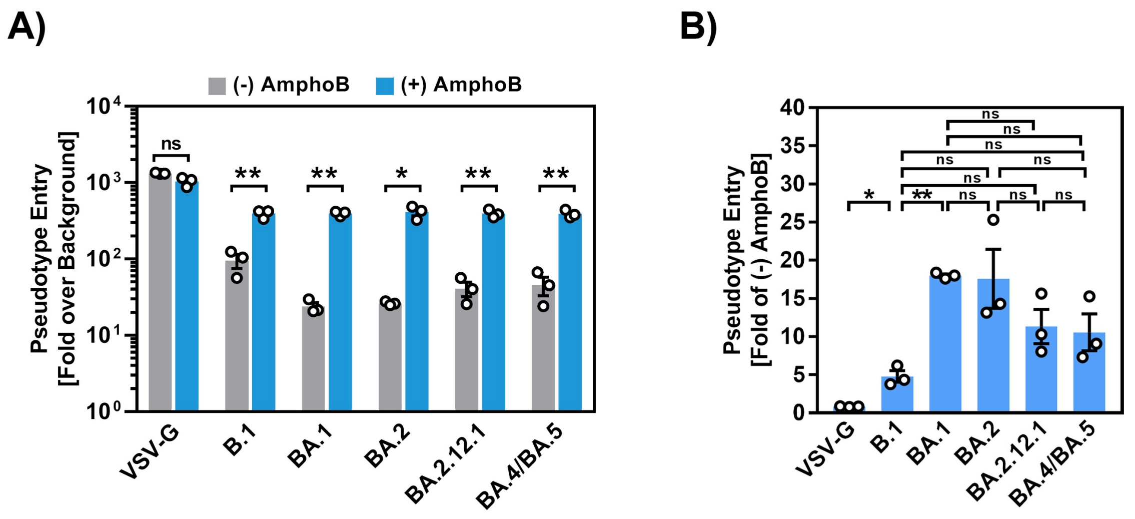

Suppl. Fig. 5 | Differential augmentation of S protein-driven Calu-3 cell entry by amphotericin B (related to the main text, discussion section).

a Calu-3 cells were preincubated (1h, 37 °C) with amphotericin b (AmphoB, 2.5 µM) before pseudoviruses were added. Pseudovirus cell entry was analyzed at 16-18 hours post inoculation. Presented are the average (mean) data from three biological replicates (each with four technical replicates), for which data were normalized against the assay background (pseudoviruses bearing no viral glycoprotein). **b** The same data were normalized against cells incubated in the absence of AmphoB (set as 1). For all panels, error bars indicate SEM and statistical significance was analyzed by two-tailed Student's t-tests with Welch correction ($p > 0.05$, not significant [ns]; $p \leq 0.05$, *; $p \leq 0.01$, **; $p \leq 0.001$, ***), see also Extended Data Table 9.

Extended Data Table 1: *P*-values for Figure 1

Panel	Experimental Groups		***P*-value**
D	293T (+ACE2)	B.1 vs. B.1.617.2	0.0238
		B.1 vs. BA.1	0.0016
		B.1 vs. BA.2	0.0151
		B.1 vs. BA.2.12.1	0.0128
		B.1 vs. BA.4/BA.5	0.4898
	A549-ACE2	B.1 vs. B.1.617.2	0.0070
		B.1 vs. BA.1	0.0047
		B.1 vs. BA.2	0.0095
		B.1 vs. BA.2.12.1	0.0100
		B.1 vs. BA.4/BA.5	0.0414

Extended Data Table 2: *P*-values for Figure 2

Panel	Experimental Groups		***P*-value**
A	B.1 vs. BA.1		0.7122
	B.1 vs. BA.2		0.7938
	B.1 vs. BA.2.12.1		0.9226
	B.1 vs. BA.4/BA.5		0.6114
	BA.4/BA.5 vs. BA.1		0.8314
	BA.4/BA.5 vs. BA.2		0.6176
	BA.4/BA.5 vs. BA.2.12.1		0.1894
B	B.1 vs. BA.1		0.0232
	B.1 vs. BA.2		0.0201
	B.1 vs. BA.2.12.1		0.0081
	B.1 vs. BA.4/BA.5		0.0001
	BA.4/BA.5 vs. BA.1		0.0004
	BA.4/BA.5 vs. BA.2		0.0012
	BA.4/BA.5 vs. BA.2.12.1		0.0009
G	293T	B.1 vs. BA.1	0.0001
		B.1 vs. BA.2	0.0001
		B.1 vs. BA.2.12.1	0.0058
		B.1 vs. BA.4/BA.5	0.0080
		BA.1 vs. BA.2	0.0300
		BA.1 vs. BA.2.12.1	0.1089
		BA.1 vs. BA.4/BA.5	0.1400
		BA.2 vs. BA.2.12.1	0.8205
		BA.2 vs. BA.4/BA.5	0.8828
		BA.2.12.1 vs. BA.4/BA.5	0.9527
	Vero	B.1 vs. BA.1	0.0001
		B.1 vs. BA.2	0.0001
		B.1 vs. BA.2.12.1	0.0001

		B.1 vs. BA.4/BA.5	0.0008
		BA.1 vs. BA.2	0.0049
		BA.1 vs. BA.2.12.1	0.0035
		BA.1 vs. BA.4/BA.5	0.0261
		BA.2 vs. BA.2.12.1	0.5843
		BA.2 vs. BA.4/BA.5	0.8563
		BA.2.12.1 vs. BA.4/BA.5	0.7854
	A549-ACE2	B.1 vs. BA.1	0.0005
		B.1 vs. BA.2	0.0001
		B.1 vs. BA.2.12.1	0.0021
		B.1 vs. BA.4/BA.5	0.0033
		BA.1 vs. BA.2	0.1839
		BA.1 vs. BA.2.12.1	0.0416
		BA.1 vs. BA.4/BA.5	0.0472
		BA.2 vs. BA.2.12.1	0.1160
		BA.2 vs. BA.4/BA.5	0.1127
		BA.2.12.1 vs. BA.4/BA.5	0.8327
	Caco-2	B.1 vs. BA.1	0.0001
		B.1 vs. BA.2	0.0001
		B.1 vs. BA.2.12.1	0.0001
		B.1 vs. BA.4/BA.5	0.0001
		BA.1 vs. BA.2	0.5445
		BA.1 vs. BA.2.12.1	0.8212
		BA.1 vs. BA.4/BA.5	0.1865
		BA.2 vs. BA.2.12.1	0.4806
		BA.2 vs. BA.4/BA.5	0.0690
		BA.2.12.1 vs. BA.4/BA.5	0.3348
	Calu-3	B.1 vs. BA.1	0.0001
		B.1 vs. BA.2	0.0001
		B.1 vs. BA.2.12.1	0.0001
		B.1 vs. BA.4/BA.5	0.0001
		BA.1 vs. BA.2	0.6973
		BA.1 vs. BA.2.12.1	0.0018
		BA.1 vs. BA.4/BA.5	0.0006
		BA.2 vs. BA.2.12.1	0.0027
		BA.2 vs. BA.4/BA.5	0.0012
		BA.2.12.1 vs. BA.4/BA.5	0.9071

Extended Data Table 3: *P*-values for Figure 3

Panel	Experimental Groups			*P*-value
A	Vero	VSV-G	No Inhibitor vs. MDL28170	0.1945
			No Inhibitor vs. Camostat	0.3731

			No Inhibitor vs. MDL28170/Camostat	0.9220
		B.1	No Inhibitor vs. MDL28170	0.0001
			No Inhibitor vs. Camostat	0.2316
			No Inhibitor vs. MDL28170/Camostat	0.0001
		BA.1	No Inhibitor vs. MDL28170	0.0001
			No Inhibitor vs. Camostat	0.2201
			No Inhibitor vs. MDL28170/Camostat	0.0001
		BA.2	No Inhibitor vs. MDL28170	0.0001
			No Inhibitor vs. Camostat	0.0075
			No Inhibitor vs. MDL28170/Camostat	0.0001
		BA.2.12.1	No Inhibitor vs. MDL28170	0.0001
			No Inhibitor vs. Camostat	0.9996
			No Inhibitor vs. MDL28170/Camostat	0.0001
		BA.4/BA.5	No Inhibitor vs. MDL28170	0.0001
			No Inhibitor vs. Camostat	0.1412
	Calu-3	VSV-G	No Inhibitor vs. MDL28170	0.9975
			No Inhibitor vs. Camostat	0.7997
			No Inhibitor vs. MDL28170/Camostat	0.3448
		B.1	No Inhibitor vs. MDL28170	0.2304
			No Inhibitor vs. Camostat	0.0001
			No Inhibitor vs. MDL28170/Camostat	0.0001
		BA.1	No Inhibitor vs. MDL28170	0.0001
			No Inhibitor vs. Camostat	0.0271
			No Inhibitor vs. MDL28170/Camostat	0.0001
		BA.2	No Inhibitor vs. MDL28170	0.0001
			No Inhibitor vs. Camostat	0.0202
			No Inhibitor vs. MDL28170/Camostat	0.0001
		BA.2.12.1	No Inhibitor vs. MDL28170	0.0001
			No Inhibitor vs. Camostat	0.0207
			No Inhibitor vs. MDL28170/Camostat	0.0001
		BA.4/BA.5	No Inhibitor vs. MDL28170	0.0001
			No Inhibitor vs. Camostat	0.0160
B	Vero / MDL28170	0.625 μM	B.1 vs. BA.1	0.0001
			B.1 vs. BA.2	0.0001
			B.1 vs. BA.2.12.1	0.0001
			B.1 vs. BA.4/BA.5	0.0001
		2.5 μM	B.1 vs. BA.1	0.0001
			B.1 vs. BA.2	0.0001
			B.1 vs. BA.2.12.1	0.0001
			B.1 vs. BA.4/BA.5	0.0001
		10 μM	B.1 vs. BA.1	0.0106
			B.1 vs. BA.2	0.0246

			B.1 vs. BA.2.12.1	0.0026
			B.1 vs. BA.4/BA.5	0.0007
		40 µM	B.1 vs. BA.1	0.9999
			B.1 vs. BA.2	0.9998
			B.1 vs. BA.2.12.1	0.9997
			B.1 vs. BA.4/BA.5	0.9997
	Calu-3 / MDL28170	0.625 µM	B.1 vs. BA.1	0.9827
			B.1 vs. BA.2	0.9997
			B.1 vs. BA.2.12.1	0.9984
			B.1 vs. BA.4/BA.5	0.7420
		2.5 µM	B.1 vs. BA.1	0.0001
			B.1 vs. BA.2	0.0001
			B.1 vs. BA.2.12.1	0.0001
			B.1 vs. BA.4/BA.5	0.0001
		10 µM	B.1 vs. BA.1	0.0001
			B.1 vs. BA.2	0.0001
			B.1 vs. BA.2.12.1	0.0001
			B.1 vs. BA.4/BA.5	0.0001
		40 µM	B.1 vs. BA.1	0.0001
			B.1 vs. BA.2	0.0001
			B.1 vs. BA.2.12.1	0.0001
			B.1 vs. BA.4/BA.5	0.0001
	Caco-2 / Camostat	0.08 µM	B.1 vs. BA.1	0.9885
			B.1 vs. BA.2	0.9971
			B.1 vs. BA.2.12.1	0.9902
			B.1 vs. BA.4/BA.5	0.8688
		0.4 µM	B.1 vs. BA.1	0.5912
			B.1 vs. BA.2	0.7783
			B.1 vs. BA.2.12.1	0.9873
			B.1 vs. BA.4/BA.5	0.9059
		2 µM	B.1 vs. BA.1	0.0439
			B.1 vs. BA.2	0.0454
			B.1 vs. BA.2.12.1	0.2729
			B.1 vs. BA.4/BA.5	0.1389
		10 µM	B.1 vs. BA.1	0.0003
			B.1 vs. BA.2	0.0008
			B.1 vs. BA.2.12.1	0.0004
			B.1 vs. BA.4/BA.5	0.0005
		50 µM	B.1 vs. BA.1	0.0001
			B.1 vs. BA.2	0.0001
			B.1 vs. BA.2.12.1	0.0001
			B.1 vs. BA.4/BA.5	0.0001

	Calu-3 / Camostat	0.08 µM	B.1 vs. BA.1	0.0029
			B.1 vs. BA.2	0.0009
			B.1 vs. BA.2.12.1	0.0030
			B.1 vs. BA.4/BA.5	0.0054
		0.4 µM	B.1 vs. BA.1	0.0001
			B.1 vs. BA.2	0.0001
			B.1 vs. BA.2.12.1	0.0001
			B.1 vs. BA.4/BA.5	0.0001
		2 µM	B.1 vs. BA.1	0.0001
			B.1 vs. BA.2	0.0001
			B.1 vs. BA.2.12.1	0.0001
			B.1 vs. BA.4/BA.5	0.0001
		10 µM	B.1 vs. BA.1	0.0001
			B.1 vs. BA.2	0.0001
			B.1 vs. BA.2.12.1	0.0001
			B.1 vs. BA.4/BA.5	0.0001
		50 µM	B.1 vs. BA.1	0.0001
			B.1 vs. BA.2	0.0001
			B.1 vs. BA.2.12.1	0.0001
			B.1 vs. BA.4/BA.5	0.0001

Extended Data Table 4: *P*-values for Figure 4

Panel	Experimental Groups			*P*-value
A	Vero	BA.2	WT vs. H69Δ/V70Δ	0.4621
			WT vs. L452R	0.2900
			WT vs. F486V	0.3366
			WT vs. R493Q	0.4019
		BA.4/BA.5	WT vs. Δ69H/ Δ70V	0.1747
			WT vs. R452L	0.1589
			WT vs. V486F	0.2680
			WT vs. Q493R	0.0939
B	Calu-3	BA.2	WT vs. H69Δ/V70Δ	0.0203
			WT vs. L452R	0.6740
			WT vs. F486V	0.9652
			WT vs. R493Q	0.4780
		BA.4/BA.5	WT vs. Δ69H/ Δ70V	0.0198
			WT vs. R452L	0.0353
			WT vs. V486F	0.9213
			WT vs. Q493R	0.0002

Extended Data Table 5: *P*-values for Figure 5

Panel	Experimental Groups		*P*-value
A	Vero	B.1 vs. BA.1	0.0654

		B.1 vs. BA.2	0.7647
		B.1 vs. BA.4	0.0319
		B.1 vs. BA.5	0.0727
		BA.1 vs. BA.2	0.0769
		BA.1 vs. BA.4	0.0169
		BA.1 vs. BA.5	0.0025
		BA.2 vs. BA.4	0.0347
		BA.2 vs. BA.5	0.0462
		BA.4 vs. BA.5	0.0012
B	Calu-3	B.1 vs. BA.1	0.0004
		B.1 vs. BA.2	0.0011
		B.1 vs. BA.4	0.0011
		B.1 vs. BA.5	0.0007
		BA.1 vs. BA.2	0.3240
		BA.1 vs. BA.4	0.2211
		BA.1 vs. BA.5	0.0002
		BA.2 vs. BA.4	0.1392
		BA.2 vs. BA.5	0.0015
		BA.4 vs. BA.5	0.0014

Extended Data Table 6: *P*-values for Figure 6

Panel	Experimental Groups			***P*-value**
C	Day 2		BA.1 vs. BA.4	0.0001
			BA.1 vs. BA.5	0.0035
			BA.4 vs. BA.5	0.0043
D	SARS-2-N	Day 2	BA.1 vs. BA.4	0.0214
			BA.1 vs. BA.5	0.0327
			BA.4 vs. BA.5	0.0207
		Day 5	BA.1 vs. BA.4	0.3348
			BA.1 vs. BA.5	0.3102
			BA.4 vs. BA.5	0.0002
E	IFN-alpha	Day 2	BA.1 vs. BA.4	0.3830
			BA.1 vs. BA.5	0.0026
			BA.4 vs. BA.5	0.0149
		Day 5	BA.1 vs. BA.4	0.8397
			BA.1 vs. BA.5	0.5202
			BA.4 vs. BA.5	0.0964
	IFN-beta	Day 2	BA.1 vs. BA.4	0.0068
			BA.1 vs. BA.5	0.1217
			BA.4 vs. BA.5	0.0369
		Day 5	BA.1 vs. BA.4	0.4131
			BA.1 vs. BA.5	0.3364

			BA.4 vs. BA.5	0.0089
	IFN-gamma	Day 2	BA.1 vs. BA.4	0.0343
			BA.1 vs. BA.5	0.0251
			BA.4 vs. BA.5	0.1642
		Day 5	BA.1 vs. BA.4	0.9961
			BA.1 vs. BA.5	0.4284
			BA.4 vs. BA.5	0.4368
	IFN-lambda	Day 2	BA.1 vs. BA.4	0.0032
			BA.1 vs. BA.5	0.0009
			BA.4 vs. BA.5	0.0008
		Day 5	BA.1 vs. BA.4	0.7408
			BA.1 vs. BA.5	0.4895
			BA.4 vs. BA.5	0.3927
	ISG15	Day 2	BA.1 vs. BA.4	0.0032
			BA.1 vs. BA.5	0.0170
			BA.4 vs. BA.5	0.2001
		Day 5	BA.1 vs. BA.4	0.0197
			BA.1 vs. BA.5	0.8768
			BA.4 vs. BA.5	0.0236
	CCL2	Day 2	BA.1 vs. BA.4	0.0090
			BA.1 vs. BA.5	0.0387
			BA.4 vs. BA.5	0.0569
		Day 5	BA.1 vs. BA.4	0.6089
			BA.1 vs. BA.5	0.6677
			BA.4 vs. BA.5	0.0059
	CCL5	Day 2	BA.1 vs. BA.4	0.0461
			BA.1 vs. BA.5	0.1270
			BA.4 vs. BA.5	0.9831
		Day 5	BA.1 vs. BA.4	0.4156
			BA.1 vs. BA.5	0.4327
			BA.4 vs. BA.5	0.6595
	TNF-alpha	Day 2	BA.1 vs. BA.4	0.0297
			BA.1 vs. BA.5	0.0006
			BA.4 vs. BA.5	0.0099
		Day 5	BA.1 vs. BA.4	0.2885
			BA.1 vs. BA.5	0.7558
			BA.4 vs. BA.5	0.0308
	IL6	Day 2	BA.1 vs. BA.4	0.0058
			BA.1 vs. BA.5	0.0165
			BA.4 vs. BA.5	0.0210
		Day 5	BA.1 vs. BA.4	0.3559
			BA.1 vs. BA.5	0.3559

			BA.4 vs. BA.5	0.7358
	CXCL10	Day 2	BA.1 vs. BA.4	0.0029
			BA.1 vs. BA.5	0.0116
			BA.4 vs. BA.5	0.0113
		Day 5	BA.1 vs. BA.4	0.0033
			BA.1 vs. BA.5	0.0005
			BA.4 vs. BA.5	0.0011

Extended Data Table 7: *P*-values for Extended Data Figure 3

Panel	Experimental Groups		*P*-value
n/a	BA.2	WT vs. H69Δ/V70Δ	0.3271
		WT vs. L452R	0.3554
		WT vs. F486V	0.4593
		WT vs. R493Q	0.3637
	BA.4/BA.5	WT vs. Δ69H/ Δ70V	0.0651
		WT vs. R452L	0.2270
		WT vs. V486F	0.7888
		WT vs. Q493R	0.8945

Extended Data Table 8: *P*-values for Extended Data Figure 4

Panel	Experimental Groups		*P*-value
B	Vero	B.1: WT vs. P681R	0.0853
		B.1.617.2: WT vs. R681P	0.7913
		BA.2 WT vs. H681R	0.9665
		BA.4/BA.5: WT vs. H681R	0.7016
	293T	B.1: WT vs. P681R	0.4852
		B.1.617.2: WT vs. R681P	0.4796
		BA.2 WT vs. H681R	0.9030
		BA.4/BA.5: WT vs. H681R	0.8143
	Caco-2	B.1: WT vs. P681R	0.4937
		B.1.617.2: WT vs. R681P	0.6298
		BA.2 WT vs. H681R	0.4162
		BA.4/BA.5: WT vs. H681R	0.4610
	Calu-3	B.1: WT vs. P681R	0.1278
		B.1.617.2: WT vs. R681P	0.8704
		BA.2 WT vs. H681R	0.6762
		BA.4/BA.5: WT vs. H681R	0.4402
C	B.1: WT vs. P681R		0.0055
	B.1.617.2: WT vs. R681P		0.0333
	BA.2 WT vs. H681R		0.1555
	BA.4/BA.5: WT vs. H681R		0.1185

Extended Data Table 9: *P*-values for Extended Data Figure 5

Panel	Experimental Groups	*P*-value
A	VSV-G: (-)AmphoB vs. (+)AmphoB	0.0684
	B.1: (-)AmphoB vs. (+)AmphoB	0.0016
	BA.1: (-)AmphoB vs. (+)AmphoB	0.0014
	BA.2: (-)AmphoB vs. (+)AmphoB	0.0142
	BA.2.12.1: (-)AmphoB vs. (+)AmphoB	0.0032
	BA.4/BA.5: (-)AmphoB vs. (+)AmphoB	0.0018
B	VSV-G vs. B.1	0.0330
	B.1 vs. BA.1	0.0015
	B.1 vs. BA.2	0.0761
	B.1 vs. BA.2.12.1	0.0895
	B.1 vs. BA.4/BA.5	0.1289
	BA.1 vs. BA.2	0.9242
	BA.1 vs. BA.2.12.1	0.0967
	BA.1 vs. BA.4/BA.5	0.0894
	BA.2 vs. BA.2.12.1	0.2522
	BA.2 vs. BA.4/BA.5	0.2123
	BA.2.12.1 vs. BA.4/BA.5	0.8262

Extended Data Table 10: Primer used in this study

Name	Sequence
pCG1 Seq F	CCTGGGCAACGTGCTGGT
pCG1 Seq R	GTCAGATGCTCAAGGGGCTTCA
SARS-2-S (BamHI) F	AAGGCCGGATCCGCCACCATGTTCGTGTTTCTGGTGCTGC
SARS-2-SΔ18 (XbaI) R	AAGGCCTCTAGACTACTTGCAGCAGCTGCCACAGC
SARS-2-S Seq-1	CAAGATCTACAGCAAGCACACC
SARS-2-S Seq-2	GTCGGCGGCAACTACAATTAC
SARS-2-S Seq-3	GGCTGTCTGATCGGAGCCGAG
SARS-2-S Seq-4	TGAGATGATCGCCCAGTACAC
SARS-2-S Seq-5	GCCATCTGCCACGACGGCAAAG
BA.2 (H69Δ/V70Δ) F	CCTGGTTCCACGCCATCTCCGGCACCAATGGCACC
BA.2 (H69Δ/V70Δ) R	GGTGCCATTGGTGCCGGAGATGGCGTGGAACCAGG
BA.2 (L452R) F	CGGCAACTACAATTACCGGTACCGGCTGTTCCGG
BA.2 (L452R) R	CCGGAACAGCCGGTACCGGTAATTGTAGTTGCCG
BA.2 (F486V) F	GTAACGGCGTGGCAGGCGTCAACTGCTACTTCCCAC
BA.2 (F486V) R	GTGGGAAGTAGCAGTTGACGCCTGCCACGCCGTTAC
BA.2 (R495Q) F	CTGCTACTTCCCACTGCAGTCCTACGGCTTTCGG
BA.2 (R495Q) R	CCGAAAGCCGTAGGACTGCAGTGGGAAGTAGCAG
BA.4/5 (Δ69H/Δ70V) F	CCTGGTTCCACGCCATCCACGTGTCCGGCACCAATGGCACC
BA.4/5 (Δ69H/Δ70V) R	GGTGCCATTGGTGCCGGACACGTGGATGGCGTGGAACCAGG
BA.4/5 (R452L) F	CGGCAACTACAATTACCTGTACCGGCTGTTCCGG
BA.4/5 (R452L) R	CCGGAACAGCCGGTACAGGTAATTGTAGTTGCCG
BA.4/5 (V486F) F	GTAACGGCGTGGCAGGCTTCAACTGCTACTTCCCAC
BA.4/5 (V486F) R	GTGGGAAGTAGCAGTTGAAGCCTGCCACGCCGTTAC

BA.4/5 (Q493R) F	GCTACTTCCCACTGCGGTCCTACGGCTTTCGG
BA.4/5 (Q493R) R	CCGAAAGCCGTAGGACCGCAGTGGGAAGTAGC
B.1 (P681R) F	GACACAGACAAACAGCCGCAGACGGGCCAGATCTG
B.1 (P681R) R	CAGATCTGGCCCGTCTGCGGCTGTTTGTCTGTGTC
B.1.617.2 (R681P) F	GACACAGACAAACAGCCCCAGACGGGCCAGATCTG
B.1.617.2 (R681P) R	CAGATCTGGCCCGTCTGGGGCTGTTTGTCTGTGTC
BA.2+BA.4/5 (H681R) F	GACACAGACAAAGAGCCGCAGACGGGCCAGATCTG
BA.2+BA.4/5 (H681R) R	CAGATCTGGCCCGTCTGCGGCTCTTTGTCTGTGTC
