# Supplementary material for: Omicron subvariant BA.5 efficiently infects lung cells
Source: Nat Commun. 2023 Jun 13;14:3500. doi: 10.1038/s41467-023-39147-4 (PMC10262933; doi:10.1038/s41467-023-39147-4)
Supplement: Supplementary file 2 — Reporting Summary [file 41467_2023_39147_MOESM2_ESM.pdf]

nature portfolio

Corresponding author(s): Markus Hoffmann
Stefan Pöhlmann

Last updated by author(s): Mar 20, 2023

Reporting Summary

Nature Portfolio wishes to improve the reproducibility of the work that we publish. This form provides structure for consistency and transparency in reporting. For further information on Nature Portfolio policies, see our Editorial Policies and the Editorial Policy Checklist.

Statistics

For all statistical analyses, confirm that the following items are present in the figure legend, table legend, main text, or Methods section.

n/a	Confirmed	
☐	☒	The exact sample size (n) for each experimental group/condition, given as a discrete number and unit of measurement
☐	☒	A statement on whether measurements were taken from distinct samples or whether the same sample was measured repeatedly
☐	☒	The statistical test(s) used AND whether they are one- or two-sided<br>*Only common tests should be described solely by name; describe more complex techniques in the Methods section.*
☐	☒	A description of all covariates tested
☐	☒	A description of any assumptions or corrections, such as tests of normality and adjustment for multiple comparisons
☐	☒	A full description of the statistical parameters including central tendency (e.g. means) or other basic estimates (e.g. regression coefficient) AND variation (e.g. standard deviation) or associated estimates of uncertainty (e.g. confidence intervals)
☐	☒	For null hypothesis testing, the test statistic (e.g. F, t, r) with confidence intervals, effect sizes, degrees of freedom and P value noted<br>*Give P values as exact values whenever suitable.*
☒	☐	For Bayesian analysis, information on the choice of priors and Markov chain Monte Carlo settings
☒	☐	For hierarchical and complex designs, identification of the appropriate level for tests and full reporting of outcomes
☒	☐	Estimates of effect sizes (e.g. Cohen's d, Pearson's r), indicating how they were calculated

Our web collection on statistics for biologists contains articles on many of the points above.

Software and code

Policy information about availability of computer code

Data collection	Hidex Sense Microplate Reader Software (version 0.5.41.0), Hidex Deutschland Vertrieb GmbH, https://www.hidex.de/; ID7000 Spectral Cell Analyzer software (version 1.1.8.18211, Sony Biotechnology, San Jose, CA, USA); ChemoStar Professional software (version v.0.3.23, Intas Science Imaging Instruments)
Data analysis	ID7000 Spectral Cell Analyzer software (version 1.1.8.18211, Sony Biotechnology, San Jose, CA, USA); ImageJ software (version 1.53C, https://imagej.nih.gov/ij/); Microsoft Excel (as part of the Microsoft Office software package, version 2019, Microsoft Corporation) and GraphPad Prism 8 version 8.4.3 (GraphPad Software) were used to analyze the data.

For manuscripts utilizing custom algorithms or software that are central to the research but not yet described in published literature, software must be made available to editors and reviewers. We strongly encourage code deposition in a community repository (e.g. GitHub). See the Nature Portfolio guidelines for submitting code & software for further information.

Data

Policy information about availability of data

All manuscripts must include a data availability statement. This statement should provide the following information, where applicable:

- Accession codes, unique identifiers, or web links for publicly available datasets
- A description of any restrictions on data availability
- For clinical datasets or third party data, please ensure that the statement adheres to our policy

The sequences of SARS-CoV-2 spike proteins were obtained from GISAID database (https://gisaid.org/). All unprocessed data generated in this study are provided in the Supplementary Information. Any additional information required to reanalyze the data reported in this paper is available on reasonable request.

Human research participants

Policy information about studies involving human research participants and Sex and Gender in Research.

Reporting on sex and gender	n/a
Population characteristics	n/a
Recruitment	n/a
Ethics oversight	n/a

Note that full information on the approval of the study protocol must also be provided in the manuscript.

Field-specific reporting

Please select the one below that is the best fit for your research. If you are not sure, read the appropriate sections before making your selection.

☒ Life sciences ☐ Behavioural & social sciences ☐ Ecological, evolutionary & environmental sciences

For a reference copy of the document with all sections, see nature.com/documents/nr-reporting-summary-flat.pdf

Life sciences study design

All studies must disclose on these points even when the disclosure is negative.

Sample size	Sample size for cell culture and animal studies was chosen to allow for statistical significance, based on previous data (Hoffmann et al, Cell, 2020, PMID: 32142651; Halfmann et al, Nature, 2022, PMID: 35062015; Barut et al, Nat Commun, 2022; PMID: 36207334). For animal welfare reasons and in accordance with the pilot study character, we only used small animal numbers.
Data exclusions	No data were excluded from the analysis.
Replication	All in vitro findings were confirmed in at lest three independent experiments. All results could be reproduced. In vivo findings were obtained from three to five biological replicates, which yielded comparable results and were analyzed within one experiment.
Randomization	Randomization of cell culture studies was not done and was not applicable. Mice and ferrets were randomly assigned to groups.
Blinding	Since all of the mouse and ferret studies were performed in the BSL3 laboratory, blinding was not possible because of biosafety considerations.

Reporting for specific materials, systems and methods

We require information from authors about some types of materials, experimental systems and methods used in many studies. Here, indicate whether each material, system or method listed is relevant to your study. If you are not sure if a list item applies to your research, read the appropriate section before selecting a response.

Materials & experimental systems

n/a	Involved in the study	
☐	☒	Antibodies
☐	☒	Eukaryotic cell lines
☒	☐	Palaeontology and archaeology
☐	☒	Animals and other organisms
☒	☐	Clinical data
☒	☐	Dual use research of concern

Methods

n/a	Involved in the study	
☒	☐	ChIP-seq
☐	☒	Flow cytometry
☒	☐	MRI-based neuroimaging

Antibodies

Antibodies used

goat anti-Human IgG (H+L) cross-adsorbed secondary antibody AlexaFlour-488-conjugated antibody (Thermo Fisher Scientific, Catalog # A-11013).

anti-SARS-CoV-2 (2019-nCoV) Spike S2 Antibody, Rabbit PAb, antigen affinity purified (Biozol, Cat: SIN-40590-T62))

anti-VSV-M [23H12] antibody (Kerafast, Cat: EB0011)

anti-rabbit antibody (goat IgG anti-rabbit IgG (H+L)-HRPO (Dianova, Cat: 111-035-003)) coupled with horseradish peroxidase

anti-mouse antibody (goat IgG anti-mouse IgG (H+L)-HRPO (Dianova, Cat: 115-035-003)) coupled with horseradish peroxidase

anti-VSV-G antibody (culture supernatant from I1-hybridoma cells; ATCC no. CRL-2700)

anti-ACE2 antibody (Sino Biological, Cat: 10108-MM36)

anti-ferret IgG FITC-conjugated secondary antibody (Bethyl, A140-108F)

Validation

The primary antibodies were validated in the following studies:
Arora et al, Int J Mol Sci. 2022, PMID: 36430535 (anti-SARS-CoV-2 (2019-nCoV), Spike S2 antibody and anti-VSV-M antibody validated for immunoblot)
Lefrancios and Lyles, Virology 1982, PMID: 6180550 (anti-VSV-G antibody validated for neutralization of VSV-G-driven cell entry)
Hoffmann et al, mBio, 2022, PMID: 35467423 (anti-ACE2 antibody validated for inhibition of SARS-CoV-2 spike protein driven entry)

Eukaryotic cell lines

Policy information about cell lines and Sex and Gender in Research

Cell line source(s)

293T (human, female, kidney; ACC-635, DSMZ; RRID: CVCL_0063),
A549 cells (human, lung; CRM-CCL-185, ATCC, RRID:CVCL_0023; kindly provided by Georg Herrler),
Vero (African green monkey kidney, female, kidney; CRL-1586, ATCC; RRID: CVCL_0574, kindly provided by Andrea Maisner)
Huh-7 (human, male, liver; JCRB Cat# JCRB0403; RRID: CVCL 0336, kindly provided by Thomas Pietschmann)
Calu-3 (human, male, lung; HTB-55, ATCC; RRID: CVCL_0609, kindly provided by Stephan Ludwig)
Caco-2 cells (human, male, colon; HTB-37, ATCC, RRID: CVCL_0025; kindly provided by Georg Herrler)
Vero-hACE2-TMPRSS2 (African green monkey kidney, female, kidney; BEI resources, NR-54970)

Authentication

Cell lines were validated using STR-typing, amplification and sequencing of a cytochrome c oxidase gene fragment, microscopic examination, and/or growth characteristics. Furthermore, mycoplasma contamination was routinely tested.

Mycoplasma contamination

All cell lines tested negative for mycoplasma contamination.

Commonly misidentified lines
(See ICLAC register)

No commonly misidentified cell lines were used.

Animals and other research organisms

Policy information about studies involving animals; ARRIVE guidelines recommended for reporting animal research, and Sex and Gender in Research

Laboratory animals

BALB/c mice (Mus musculus), 6–8 weeks of age
Ferrets (Mustela putorius furo), 2.1 to 3.2 years of age

Wild animals

No wild animals were used in this study.

Reporting on sex

Female animals were used. Animal sex is not expected to impact study results.

Field-collected samples	No field collected samples were used in this study.
Ethics oversight	All mouse studies were approved by the University of Iowa Animal Care and Use Committee and meet stipulations of the Guide for the Care and Use of Laboratory Animals.<br>The ferret infection study was evaluated by the responsible ethics committee of the State Office of Agriculture, Food Safety, and Fishery in Mecklenburg–Western Pomerania (LALLF M-V) and gained governmental approval under the registration number LVL MV TSD/7221.3-2-005/21

Note that full information on the approval of the study protocol must also be provided in the manuscript.

Flow Cytometry

Plots

Confirm that:

☒ The axis labels state the marker and fluorochrome used (e.g. CD4-FITC).

☒ The axis scales are clearly visible. Include numbers along axes only for bottom left plot of group (a 'group' is an analysis of identical markers).

☒ All plots are contour plots with outliers or pseudocolor plots.

☒ A numerical value for number of cells or percentage (with statistics) is provided.

Methodology

Sample preparation	The cells were rinsed in PBS containing 1% bovine serum albumin (BSA, PBS-B) and pelleted. The cell pellets were then resuspended in 250 µl PBS-B containing different concentrations of soluble solACE2-Fc (Bio-Techne) and rotated for 60 minutes at 4 °C using a Rotospin test tube rotator disk (IKA). Cells were pelleted, resuspended in 250 µl PBS-B containing anti-human AlexaFluor-488-conjugated antibody (1:200; Thermo Fisher Scientific), and rotated for 60 minutes at 4 °C. Finally, the cells were washed in PBS-B, fixed for 30 minutes at room temperature in a 1 % paraformaldehyde solution, washed again, and resuspended in 100 µl PBS-B.
Instrument	ID7000 Spectral Cell Analyzer (Sony Biotechnology, San Jose, CA, USA)
Software	ID7000 software
Cell population abundance	Does not apply since a cell line was analyzed.
Gating strategy	We gated for single cells and then analyzed fluorescence of this cell population, as shown in Suppl. Fig. 1.

☒ Tick this box to confirm that a figure exemplifying the gating strategy is provided in the Supplementary Information.
